# Clinical profile and outcome of patients with acute kidney injury in the Emergency Department of a teaching hospital in Ethiopia

**DOI:** 10.1016/j.afjem.2025.100926

**Published:** 2025-11-21

**Authors:** Berihu Assefa Berhe, Benyam Bahta Gebrehiwot, Frehiwot Worku Senbeta, Dirijit Mamo Alemu, Menbeu Sultan Mohammed, Mohammed Kalifa Nuguse, Yemane Gebremedhin Tesfay

**Affiliations:** a Department of Emergency Medicine and Critical Care, Mekelle University College of Health Sciences, Ayder Comprehensive Specialized Hospital, Mekelle, Tigray, Ethiopia; b Department of Emergency Medicine and Critical Care, Saint Paul’s Hospital Millennium Medical College, Addis Ababa, Ethiopia; c Department of Public Health, Saint Paul’s Hospital Millennium Medical College, Addis Ababa, Ethiopia

**Keywords:** Acute kidney injury, Clinical profile, Outcome, Emergency Department, Ethiopia

## Abstract

**Background:**

Acute Kidney Injury (AKI) affects 13.3 million people annually, with 85% in low resource settings. This study aimed to identify AKI profiles and outcomes in Ethiopia's largest teaching hospital's emergency department, focusing on targeted therapy and preventative measures.

**Method and Materials:**

A cross-sectional study was conducted at an Ethiopian teaching hospital's Emergency Department from June 1, 2021, to June 1, 2022. Descriptive statistics and binary logistic regression were analyzed. A paired samples T-test was used to compare patient findings.

**Results:**

Among the 222 AKI patients included in the study, 110 (49.5 %) were males, and 112 (50.5 %) were females. The mean age of presentation was 48±18 years old. Most patients were from Addis Ababa (41.4 %) and the Oromia region (40.5 %). The most common causes of AKI were infections (26.2 %), acute glomerulonephritis (20.4 %), volume depletion (18.5 %), and obstructive uropathy (16.6 %). Uremic encephalopathy, infection, malignancy, and a low initial Glasgow Coma Scale significantly contributed to unfavorable outcomes. The presence of nephrotoxic antibiotics, infection, and hyponatremia significantly contributed to the admission rate to the wards and intensive care unit.

**Conclusion:**

In conclusion, infection is the dominant cause and mortality predictor of AKI. The majority of patients with infections were sepsis (78.1 %), pyelonephritis (11.4 %), and pneumonia (10.3 %). Early initiation of antibiotics in the Emergency Department contributes to improved outcomes.

## Highlights

• AKI is underreported cause of morbidity and mortality in African emergency care.
• This study reveals clinical traits, causes & outcomes in Ethiopian hospital.
• Findings stress early AKI care, diagnostics & tailored strategies in Africa.
• Data supports AKI policy, care & research across sub-Saharan Africa settings.

## African relevance

• Acute kidney injury (AKI) is a significant yet underreported cause of morbidity and mortality in African emergency departments, where diagnostic and therapeutic resources are often limited.
• This study provides valuable insight into the clinical profile, common etiologies, and outcomes of AKI in an Ethiopian tertiary hospital setting.
• The findings highlight context-specific challenges and emphasize the need for early recognition, improved diagnostic capacity, and resource-appropriate management strategies in low-resource emergency care settings across Africa.
• The data can inform national and regional health policies aimed at improving AKI outcomes and guide future research across similar healthcare environments in sub-Saharan Africa.

## Introduction

Acute kidney injury (AKI) is defined as a decline in renal function over hours or days, resulting in the accumulation of toxic wastes and the loss of internal homeostasis. AKI is defined according to the Kidney Disease Improving Global Outcomes (KDIGO) 2012 criteria as an increase in serum creatinine (Scr) by ≥ 0.3 mg/dl within 48 h, or an increase in Scr to ≥ 1.5 times baseline within the prior 7 days, and urine volume < 0.5 mL/ kg/h for 6 h [1]. The Acute Kidney Injury Network (AKIN) proposed in 2007, that AKI is a decline in kidney function during 48 h as demonstrated by an increase in serum creatinine of more than 0.3 mg/dl, or an increase in serum creatinine of more than 50 %, or the development of oliguria [2,3].

AKI affects approximately 13.3 million individuals globally per year. An estimated 85 % of those affected live in low and middle income settings. AKI is thought to contribute to about 1.7 million deaths every year [4,5]. Acute kidney injury (AKI) is a challenging problem in Africa because of the high burden of infectious diseases, the late presentation of patients to healthcare facilities, and the lack of resources to support patients with diagnosed AKI [6,7].

The clinical importance of AKI is exemplified by data showing a consistent association with increased long-term risk of poor outcomes, including death, incident chronic kidney disease (CKD), and greater utilization of health resources. AKI is a common complication of critical illness, with some research showing that as many as 1 in 5 adults experience AKI per hospital admission [8].

The International Society of Nephrology conducted a "Global Snapshot" about AKI in 2014, where most cases (45 %) were from low and lower-middle-income countries, with the proposed reasons for the high incidence being a late presentation of patients to tertiary centers, underreporting, and a reduced capacity to provide intensive care to severely ill patients [9]. The main objective of this study was to identify the clinical profile of patients with AKI in the Emergency Department (ED) and to assess the treatment outcome in the largest teaching hospital in Ethiopia.

## Methods and materials

This study was conducted at the ED of the largest teaching hospital and the only kidney transplant center in Ethiopia, St. Paul`s Hospital Millennium Medical College (SPHMMC) [10,11]. A hospital-based cross-sectional study was conducted over one year to analyze AKI cases in the emergency department from June 1, 2021, to June 1, 2022.

All patients seen in the ED of SPHMMC during the study period were the source population, comprising 504 patients to include 222 patients with AKI.

Patients presented to the emergency department of SPHMMC and were diagnosed with acute kidney injury according to the Kidney Disease Improving Global Outcomes (KDIGO) definition, fulfilling the following inclusion criteria: were included: increase in serum creatinine by ≥0.3 mg/dL within 48 h; or increase in serum creatinine to ≥1.5 times baseline, known or presumed to have occurred within the prior 7 days; or urine output <0.5 mL/kg/h for 6 h hours.

Sample size was determined using a single population proportion formula, with a 5 % margin of error, and a 30 % prevalence of AKI patients, resulting in a final sample size of 222 patients, which were included conveniently [[12], [13]]. The study collected data from patients with acute kidney injuries using a structured checklist in Google Forms. The data included different important variables prepared from literature reviews and guidelines on AKI and filled by SPHMMC emergency and critical care medicine resident physicians. A 10 % pretest was conducted a week before data collection, with modifications made to the checklist. Data collectors received training, and daily double-checks ensured consistency and completeness. The data was processed using Microsoft Excel and IBM SPSS version 29 for data entry, analysis, and identification of missing values. Descriptive statistics were used to calculate demographic and clinical features, and relationships between variables were identified using paired binary and *t*-tests.

St. Paul's Hospital Millennium Medical College Institutional Review Board in Addis Ababa provided ethical clearance and permission for this study with Ref. No. pm23/11. No personal identifiers were used in the data collection to ensure confidentiality. The need for informed consent and publication was waived by SPHMMC IRB due to the retrospective nature of data collection.

## Results

Of the 222 AKI patients identified, 112 (50.3 %) were female. The age range of the patients was 15–90 years old. More than two-thirds (77.5 %) of patients were referred from government health facilities. All self-referred patients (9.9 %) were from the Addis Ababa and Oromia regions. See Table 1.

**Table 1:** Demographic characteristics of Acute Kidney Injury patients presented to the Emergency Department.

Demographic characteristics	Sub-variables	Frequency	Percent
**Age groups**	14–24	24	10.8
	25–34	29	13.1
	35–44	44	19.8
	45–54	39	17.6
	55–64	37	16.7
	>64	49	22.1
**Residence**	Addis Ababa	92	41.4
	Amhara	16	7.2
	Oromia	90	40.5
	SNNPR	18	8.1
	Others	6	2.7
**Patient referred from**	Self-referral (home)	22	9.9
	Government health facility	172	77.5
	Private health facility	28	12.6
**Gender**	Male	110	49.5
	Female	112	50.5

**AKI-** Acute Kidney Injury, **ED-** Emergency Department, **SNNPR**- Southern Nations, Nationalities and Peoples Region.

The majority of patients, 141 (63.5 %), were triaged to yellow-green, 60 (27.3 %) were triaged to orange, and 21 (9.46 %) were triaged to the red area of the emergency department according to the South African Triage Scale (SATS) [21]. Only 112 (50.3 %) of patients had a normal blood pressure range. Some 26 patients (11.6 %) and 72 patients (32.4 %) were in the hypotensive and hypertensive range, respectively. Only 12 (5.3 %) patients presented with hypertensive crises. More than three-fourths of patients presented with normal temperature, whereas 13.4 % had a record of fever on presentation. Most patients had random blood sugar (RBS) of 140–180 (58.9 %), and 24.7 % of patients with AKI presented with RBS of greater than 180 mg/dl. Only 1.3 % of patients had hypoglycemia on presentation to the emergency department.

The most common premorbid condition identified was hypertension (27.2 %), followed by malignancy (24.79 %) and diabetes mellitus (19 %). Heart failure and Human Immunodeficiency Virus (HIV) account for 14 % and 8.2 %, respectively.

The commonest presenting features of AKI were shortness of breath 46 (20.7 %), followed by oedema 44 (19.8 %), and decreased urine output 43 (19.3 %). One hundred and eighty (81 %) of the patients were fully conscious (GCS- 15/15), and 40 (18 %) patients had moderately altered mental status (GCS of 9–14). The remaining two (0.9 %) of patients presented with coma (GCS ≤ 8) to the ED. Most of the patients (81.3 %) had a duration of complaint of two days to two weeks, and 13.6 % had more than two weeks duration of complaint.

The commonest causes of AKI were infection 71 (26.2 %), fulfilling the diagnosis of sepsis in 78.1 % from sources of pyelonephritis, in 11.4 % of patients, followed by pneumonia, in 10.3 %. Acute glomerulonephritis was observed in 53 (20.4 %), which was not a biopsy diagnosis based on signs and symptoms and urine analysis as documented by the treating physician. The most commonly identified nephrotoxic drugs were antibiotics in 50 patients (28.4 %), mainly vancomycin and gentamicin, and immunosuppressive drugs, comprising most anti-cancer medications, such as cisplatin, in 49 patients (17.8 %). Nonsteroidal anti-inflammatory drugs (NSAIDs), diuretics, and angiotensin-converting enzyme (ACEI) were incriminated in 34 (19.3 %), 29 (16.5 %), and 14 (8.0 %) of patients.

Comparison of laboratory values during the initial and discharge periods was performed to assess the implications of monitoring the progression or resolution of kidney dysfunction and the impact of treatment during the ED stay. One hundred eighty-eight (84.5 %) patients had elevated initial creatinine, with a good decrement upon discharge from a mean of 4.54±4.73 to 3.6 ± 3.81 mg/dL. And 137 (61.7 %) patients had normal range potassium. One hundred and three (46.4 %) patients presented with hyponatremia. See Tables 2 & 3 below.

**Table 2:** Initial Investigation of Acute Kidney Injury patients presented to the Emergency Department.

Investigation results	Male (*n* = 110)	Female (*n* = 112)	Total (*n* = 222)
**Hemoglobin level (Hgb)**			
Normal	71 (31.9 %)	55 (24.7 %)	126 (56.6 %)
Mild anemia	14 (6.3 %)	22 (9.9 %)	36 (16.2 %)
Moderate anemia	21 (9.4 %)	28 (12.6)	49 (22.0 %)
Severe anemia	4 (1.8 %)	7 (3.1 %)	11 (4.9 %)
**White Blood Cell Count (WBC)**			
Normal range	70 (31.5 %)	67 (30.1 %)	137 (61.6 %)
Leukopenia	4 (1.8 %)	6 (2.7 %)	10 (4.5 %)
Leukocytosis	36 (16.2 %)	39 (17.5 %	75 (33.7 %)
**Platelet**			
Normal	48 (21.6 %)	58 (26.1 %)	106 (47.7 %)
Thrombocytopenia	55 (24.7 %)	47 (21.1 %)	102 (45.8 %)
Thrombocytosis	7 (3.1 %)	7 (3.1 %)	14 (6.2 %)
**Creatinine (Cr.)**			
Normal	17 (7.65 %)	17 (7.65 %)	34 (15.3 %)
Elevated	93 (41.9 %)	95 (42.8 %)	188 (84.7 %)
**Serum Potassium (*****K*****+**>**)**			
Normal	68 (30.6 %)	69 (31.0 %)	137 (61.7 %)
Hypokalemia	11 (4.9 %)	10 (4.5 %)	21 (9.4 %)
Hyperkalemia	31 (13.9 %)	33 (14.8 %)	64 (28.7 %)
**Serum Sodium (Na+)**			
Normal	53 (23.8 %)	51 (22.9 %)	104 (46.8 %)
Hyponatremia	50 (22.8 %)	53 (23.8 %)	103 (46.6 %)
Hypernatremia	7 (3.1 %)	8 (3.6 %)	15 (6.8 %)

**Table 3:** Laboratory values of Acute Kidney Injury patients in the study.

Laboratory Results		Mean	Standard Deviation
White Blood Cell Count (WBC) in *cells/μL*	Initial	12,219.50	10,385.29
	Discharge	13,689.46	21,444.76
Hemoglobin (Hgb) *g/dL*	Initial	11.87	2.88
	Discharge	11.46	2.66
Platelets (PLT) Count *cells/μL*	Initial	263,936.94	123,036.67
	Discharge	273,783.33	153,007.08
Blood Urea Nitrogen (BUN) *mg/dL*	Initial	108.63	95.48
	Discharge	92.50	84.93
Serum Creatinine (Cr.) *mg/dL*	Initial	4.55	4.73
	Discharge	3.60	3.81
Serum Potassium (*K*+) mEq/L	Initial	4.75	1.19
	Discharge	4.46	0.94
Serum Sodium (Na+) mEq/L	Initial	134.50	8.95
	Discharge	136.03	7.50

More than half (62.1 %) received conservative management of drugs, fluids, and urinary catheterization, and 16 % of patients received additional operative management for obstructive causes. Most of the AKI patients (68.0 %) stayed in the ED for 1–5 days, 30.1 % stayed more than 5 days & 1.8 % of patients stayed less than a day. Hemodialysis was given to 52 (23.4 %) of the patients. The identified indications for dialysis were refractory fluid overload (57.6 %), uremic complications (30.7 %), and refractory hyperkalemia (11.5 %). The most common complication identified in the ED was hyponatremia, accounting for 32.5 %, hyperkalemia at 22.7 %, pulmonary edema at 18.7 %, uremic gastropathy at 12.1 %, and other complications at 7.4 %, and uremic encephalopathy at 6.3 %. Almost half of the patients (50.9 %) were admitted to the ward or ICU, one-third (33.3 %) were discharged home, and twenty-two (9.9 %) patients with AKI died in the ED with refractory septic shock as an immediate cause of death in 72.7 %, hypoxia in 22.7 %, and massive pulmonary embolism in 4.5 % of patients.

A paired *t*-test was been conducted to evaluate the impact of AKI treatment on the change in laboratory values during emergency admission and upon discharge from the ED. The result showed a significant decrease in creatinine from 4.5 mg/dl (SD 4.7) to 3.6 mg/dl (SD 3.8), t-value of 4.3, and p-value of < 0.000. The mean decrement in creatinine was 0.937 mg/dl with a 95 % confidence interval ranging from 0.51 to 1.36. There was also a significant change in mean sodium increase from 134 (SD 8.9) to 36 (SD 7.5), t-value of −2.7, and p-value of 0.007. The initial sodium increased with a mean value of 1.5, with a 95 % confidence interval ranging from 2.6 to 0.4.

Regarding the WBC count, there was an increase from 12,219 (SD. 10,385) to 13,689 (SD. 21,444), t-value of −1.06, and p-value of 0.290 with 95 % CI ranging from 4203 to 1263. But, it was not a significant change as the p-value is greater than 0.05. On the other hand, there was a significant change in hemoglobin decrement by a mean value of 0.4 mg/dl from the admission value (t-value 3.2, and p-value 0.002 at a 95 % CI), ranging from 0.15 to 0.65. There was also a significant change in potassium increment with a mean value of 0.2896, t-value of 4.2, and p-value of 0.000 with a 95 % CI ranging from 0.12 to 0.42. See Table below. Table 4

**Table 4:** Paired T-test of laboratory values of Acute Kidney Injury patients enrolled.

Paired Differences of initial and discharge laboratory values								
Laboratory Variables	Mean	Std. Deviation	Std. Error Mean	95 % Confidence Interval (CI) Difference		t-value Difference	Significance (2-tailed)	
				Lower	Upper			
WBC	−1469.955	20,667.410	1387.106	−4203.602	1263.692	−1.060	221	.290
Hgb	.4072	1.8815	.1263	.1584	.6561	3.225*	221	.001*
PLT	−9846.396	145,788.517	9784.685	−29,129.62	9436.832	−1.006	221	.315
BUN	16.128	79.504	5.336	5.613	26.644	3.023*	221	.003*
Cr.	.93770	3.21683	.21590	.51222	1.36319	4.343*	221	.000*
*K*+	.2896	1.0242	.0687	.1541	.4251	4.213*	221	.000*
Na+	−1.533	8.436	.566	−2.649	−0.418	−2.708*	221	.007*

⁎ significant association: P-value < 0.05, t-value > ±2.

Binary logistic regression (95 % CI with p-value of < 0.05) was implemented to determine the independent predictors of mortality among AKI patients. Overall, AKI patients' mortality was significantly correlated with the presence of uremic encephalopathy, infection, nephrotoxic-antibiotics, malignancy, and low initial GCS. It was also correlated with discharge potassium level and pulmonary edema solely before adjustment of the odds ratio.

Admission to the ICU and ward from the ED was significantly affected by those who were taking nephrotoxic antibiotics (AOR 0.24 (CI: 0.11–0.55), *p* = 0.001 — inverse association), patients with hyponatremia (OR: 2.1 (CI: 1.25–3.6), *P* = 0.005). Also, acute glomerulonephritis (OR: 0.39 (CI: 0.20–0.75), *P* = 0.005) indicates a protective association or lower likelihood of admission, possibly due to early detection or milder presentation. Still significant, reinforcing its role in influencing admission decisions (Adjusted OR: 0.45 (CI: 0.22–0.91), *P* = 0.027), and infection (AOR 2.1 (CI: 1.04–4.3), *p* = 0.039). See Table 5 below.

**Table 5:** Binary logistic regression analysis between different factors and death.

Death				
Associated Factors	COR (95 %CI))	P-value	AOR (95 %CI	P-Value
Uremic encephalopathy	12.9 (4.6–36)	0.000*	17 (5–60)	0.000*
Pulmonary edema	2.6 (1.1–6.5)	0.03	2.1 (0.7–6)	0.167
Infection	4 (1.7–11.2)	0.001*	3.2 (1.2–8.5)	0.017*
Discharge Potassium	1.6 (1.08–2.5)	0.002	1.54 (0.88–2.6)	0.126
Nephrotoxic antibiotics	2.6 (1.074–8.7)	0.035*	2.7 (1.038–7.29)	0.042*
Malignancy	4.1 (1.4–11.9)	0.007*	3.5 (1.3–9.6)	0.012*
Initial Glasgow Coma Scale	4.7 (2.06–10.9)	0.000*	3.7 (1.5–9.06)	0.003*
Admission to inpatient management				
Nephrotoxic antibiotics	2.5(1.2–4.8)	0.007*	0.24(0.11–0.55)	0.001*
Infection	2.3(1.1–4.3	0.014*	2.1(1.04–4.3)	0.039*
Hyponatremia	2.1(1.25–3.6)	0.005*	0.45(0.25–0.83)	0.011*
Acute glomerulonephritis	0.39(0.20–0.75)	0.005*	0.45(0.22–0.91)	0.027*

⁎ significant association: P-value < 0.05.

## Discussion

This study elucidated the referral status, presenting vital signs, triage categories, main presenting complaints, causes, complications, the practice of management, and outcomes of patients with AKI. The presentation of the patients at a younger age was consistent with results from other African countries as well as those done in Ethiopia [6,[14], [15], [16]]. Forty-nine patients (22.1 %) were over the age of sixty-four in our study, as opposed to the study conducted by Jimma Medical Center, which included 28 % of AKI patients in this age range [18].

The most frequent comorbid conditions in our study were more or less similar to research conducted in South Africa in 2017. However, in Uganda, HIV accounts for 60 % of all comorbid conditions, which might be due to lower triggering factors to screen HIV in AKI patients, though HIV, its complications, and the medications taken could cause significant renal derangement [8]. The most presenting complaint of AKI patients to the ED was consistent with a study done in Tanzania [18].

The most common causes of AKI identified in our study were infections, acute glomerulonephritis, and hypovolemia. In a previous study done in the same hospital, the commonest cause identified was hypovolemia. Obstructive uropathy accounts for the fourth most common cause of AKI in our study, which is similar to studies done in the same city and with the same setup. However, the most common cause of obstructive uropathy identified in our patients was nephrolithiasis rather than cervical cancer in the comparative studies done in the same study [6,15,18].

Regarding AKI-associated complications, hyponatremia, hyperkalemia, and pulmonary edema were the most common. Though our findings with hyperkalemia and pulmonary edema were similar in other studies [[17], [18]]. Hyponatremia was a different finding in our study. Our findings regarding management were more or less similar to previous studies done in another hospital in the same city and a study done in Tanzania [12,18].

The most common indications for dialysis were refractory fluid overload, uremic complications, and hyperkalemia. As compared to other studies done in a similar city and the same setup, uremic encephalopathy was the most common indication for dialysis, followed by hyperkalemia and fluid overload. Metabolic acidosis could not be diagnosed in our hospital, as there was no arterial blood gas analysis [12]. Around 84 % of our patients had community-acquired AKI, similar to a study done in Ghana [16]. The mortality rate of 9.9 % in our study was much lower than the data from a global meta-analysis of studies done across the globe, but was for dialysis-requiring AKI, which was 49.4 %, and also in Tanzania, 39 % [[18], [19], [20]].

The same higher mortality of AKI has been observed in a study done in TASH, which is a tertiary teaching hospital, in the same capital city [12]. It may be due to the inclusion of AKI in CKD patients and treatment-related mortalities [15]. However, our result was similar to a meta-analysis done in Ghana in the range of 11.5–43.5 % [18]. In our current study, uremic encephalopathy, infection, malignancy, serum potassium, and low initial GCS significantly contributed to death, and in a study done in Jimma medical center, the duration of AKI, the severity of AKI, and hyperkalemia were associated with mortality [18]. A study done in Sudan showed that increased age, severity of AKI, presence of sepsis, and liver disease contributed to increased mortality [14].

## Conclusions

Most low- and middle-income countries showed similarities in the AKI profile, and were primarily found to be a community-acquired condition in most of our patients. Infections, acute glomerulonephritis, and hypovolemia were the most frequent causes of AKI. Significant mortality predictors included uremic encephalopathy, infection, malignancy, and low GCS at presentation.

Health care providers should focus on the prevention strategies for the most common contributors to AKI development, mortality, complications, and causes of admission to decrease the health institutions' burden of care. ED staff should prioritize early identification of infection-related AKI, initiate empiric antibiotic therapy promptly, screen for nephrotoxic drug exposure, monitor electrolytes aggressively, use the Glasgow Coma Scale as a prognostic tool, strengthen AKI triage and referral pathways, expand dialysis access and criteria, and educate staff on AKI risk factors and outcomes. Infection is the leading cause of AKI (26.2 %) and a major predictor of mortality. Implementing rapid screening protocols for infection signs and using triage tools for immediate physician review can help. Developing standing orders for empiric antibiotics and integrating a nephrotoxic drug alert system into triage or electronic medical records can improve outcomes.

## Dissemination of results

The results of the study were shared with staff members of the Emergency and Critical Care Medicine department of St. Paul`s Hospital Millennium Medical College (SPHMMC). The results will also be presented to the medical or scientific community at different national and international meetings.

## CRediT authorship contribution statement

**Berihu Assefa Berhe:** Data curation, Writing – original draft. **Benyam Bahta Gebrehiwot:** Conceptualization, Methodology, Software. **Frehiwot Worku Senbeta:** Visualization, Investigation. **Dirijit Mamo Alemu:** Software, Validation. **Menbeu Sultan Mohammed:** Supervision. **Mohammed Kalifa Nuguse:** Resources, Software, Validation. **Yemane Gebremedhin Tesfay:** Writing – review & editing, Validation, Supervision, Resources.

## Declaration of competing interest

The authors declared no conflicts of interest.
